# Role of robot-assisted laparoscopy in deep infiltrating endometriosis with bowel involvement: a systematic review and application of the IDEAL framework

**DOI:** 10.1007/s00384-024-04669-w

**Published:** 2024-06-26

**Authors:** Hwa Ian Ong, Nastassia Shulman, Patrick Nugraha, Stephen Wrenn, Deirdre Nally, Colin Peirce, Uzma Mahmood, Jacob McCormick, David Proud, Satish Warrier, Christina Fleming, Helen Mohan

**Affiliations:** 1 University of Melbourne, Melbourne, Australia; 2 Department of Colorectal Surgery, Austin Health, Melbourne, Australia; 3 Department of Colorectal Surgery, University Hospital Limerick, Limerick, Ireland; 4 Department of Gynaecology Surgery, University Hospital Limerick, Limerick, Ireland; 5 Peter MacCallum Cancer Center, Melbourne, Australia

**Keywords:** Deep infiltrating endometriosis, Robotic surgery, Bowel surgery, Laparoscopy, Surgical treatment, Minimally invasive surgery

## Abstract

**Aims:**

This review aims to evaluate the feasibility of robot-assisted laparoscopic surgery (RALS) as an alternative to standard laparoscopic surgery (SLS) for the treatment of bowel deep-infiltrative endometriosis. Additionally, it aims to provide guidance for future study design, by gaining insight into the current state of research, in accordance with the IDEAL framework.

**Method:**

A systematic review was conducted to identify relevant studies on RALS for bowel deep infiltrating endometriosis in Medline, Embase, Cochrane Library and PubMed databases up to August 2023 and reported in keeping with PRISMA guidelines. The study was registered with PROSPERO Registration: CRD42022308611

**Results:**

Eleven primary studies were identified, encompassing 364 RALS patients and 83 SLS patients, from which surgical details, operative and postoperative outcomes were extracted. In the RALS group, mean operating time was longer (235 ± 112 min) than in the standard laparoscopy group (171 ± 76 min) (*p* < 0.01). Patients in the RALS group experienced a shorter hospital stay (5.3 ± 3.5 days vs. 7.3 ± 4.1 days) (*p* < 0.01), and appeared to have fewer postoperative complications compared to standard laparoscopy. Research evidence for RALS in bowel DE is at an IDEAL Stage 2B of development.

**Conclusion:**

RALS is a safe and feasible alternative to standard laparoscopy for bowel endometriosis treatment, with a shorter overall length of stay despite longer operating times. Further robust randomized trials recommended to delineate other potential advantages of RALS.

**Supplementary Information:**

The online version contains supplementary material available at 10.1007/s00384-024-04669-w.

## Introduction

Endometriosis is a chronic condition characterized by the presence of endometrial-like tissue outside the uterus, affecting around 10% of women of childbearing age [1, 2]. The ectopic endometrial tissue responds to hormonal fluctuations during the menstrual cycle, leading to pain, inflammation and adhesion formation in affected organs [3]. Clinical presentations can vary, but women with endometriosis commonly present with pelvic pain, dysmenorrhea and infertility [1].

Deep infiltrative endometriosis (DE) [4] is defined as lesions with ≥ 5-mm penetration into the peritoneum or lesions that invade the muscularis propria of surrounding viscera, affecting more than 20% of women with said condition [2, 5]. Among the known sites of DE, bowel involvement is reported in 5–12% of women with endometriosis and can be associated with additional symptoms such as abdominal pain, hematochezia, and dyschezia [6]. A substantial subset of this, ranging from 70 to 93%, exhibits rectosigmoid involvement [5, 7].

The primary approach for managing symptoms of bowel deep infiltrating endometriosis [4] involves medical therapy, including non-steroidal anti-inflammatory drugs, oral contraceptives and progestins [6, 8]. Once medical options have been exhausted, surgical removal has been considered as the next step in the treatment pathway, with standard laparoscopic surgery (SLS) being the current standard approach [1, 6, 9]. Various surgical techniques have been described for treating bowel DE, including rectal shaves, discoid excisions or segmental resections.

Each of the above techniques involves an increasing degree of surgical complexity, exposing patients to the risk of more significant complications. Therefore, the selection of the surgical technique depends on the extent, length and depth of bowel involvement and may vary depending on preferences and experience of the surgeon involved [6, 9–11].

Robot-assisted laparoscopic surgery (RALS) has been shown to be a viable alternative to conventional laparoscopy, providing technical advantages such as improved dexterity and ergonomics, increased range of motion, three-dimensional visualization and elimination of tremors [1, 9, 11]. However, arguments for the feasibility of RALS over standard laparoscopy in the treatment of symptomatic Bowel DE remains limited in the literature, confounded with discrepancies in outcomes reporting.

The main aim of this review is to collate and examine the existing data on RALS in the treatment of bowel DE among symptomatic patients. This review aims to assess surgical procedural details, operative parameters and postoperative outcomes to provide insights into the feasibility of the RALS approach for this population.

Additionally, this review also aims to evaluate the developmental status of the RALS technique based on the IDEAL framework. The framework describes five stages—Idea, Development, Exploration, Assessment and Long-term study—in the evaluation of emerging surgical techniques, determined by the types of studies already published on said technique [12–14]. Understanding the current developmental stage allows researchers to identify gaps in the current evidence base and guides the direction of future research in this space.

## Methods

This review was designed according to PRISMA guidelines [15] and was submitted to the PROSPERO database, CRD42022308611. The search was last accessed in August 2023, of all publications in Medline, Embase, PubMed and Cochrane Database, reporting on the outcomes of robot-assisted laparoscopic interventions for bowel DE. Table 1 shows the key terms and Boolean operators used for the search in the title and abstract fields. A manual search for relevant studies was also carried out to identify additional studies.

**Table 1:** Keyword search strategy with combined search terms

#	Keyword search strategy
1	endometriosis
2	(“colon” or “colorectal” or “rectum” or “bowel”)
3	(“colorectal surgery” or “colorectal resection” or “rectal shave” or “discoid excision” or “segmental resection”)
4	(“robot*” or “laparoscop*” or “robot-assisted surgery”)
5	1 and 2
6	1 and 3
7	5 or 6
8	4 and 7

### Inclusion and exclusion criteria

Primary studies published up to August 2023 were considered for inclusion to ensure the relevance and timeliness of the findings. This review considered studies discussing RALS procedures for treating bowel DE, including rectal shaves, discoid excisions and segmental resections. These studies can either be non-comparative (RALS only) or comparative studies (RALS vs. SLS). The review population was restricted to symptomatic patients diagnosed with bowel DE through clinical evaluations and/or imaging.

Studies with an emphasis on standard laparoscopic or laparotomy interventions only and those examining DE which did not involve bowel were excluded from consideration. Additionally, literature reviews, conference abstracts, single-case reports and studies not providing outcomes of interest were excluded.

### Study characteristics and outcomes of interest

The assessment of the study characteristics included variables such as authorship, publication year, geographical origin, study design, sample size, procedures of interest and key findings. Furthermore, outcomes from each study were extracted using a prospectively designed collection sheet, encompassing technical details, intraoperative parameters and postoperative outcomes.

### Risk of bias assessment

The methodological quality and risk of bias in included studies were assessed using the Joanna-Briggs Institute (JBI) appraisal tools for case series [16] and cohort studies [17], summarised in Table 8.

### Data extraction and statistical analysis

Two independent reviewers (PN, NS) conducted title and abstract screening, full-text inclusion and data extraction, with disputes settled with another reviewer (HO) if consensus was not reached. Data were summarized in a narrative synthesis and the chi-squared test, Fisher’s exact test and Student’s *t*-test were used for the comparison of categorical and numerical variables, where appropriate. In cases where variables were presented in the form of median/range, the mean/standard deviation were estimated employing methods outlined by Luo et al. [18] and Wan et al. [19]. All reported *p*-values were two-sided, and a significant difference was noted when *p* < 0.05. Statistical analysis was performed using commercially available software.

## Results

**Fig. 1:**
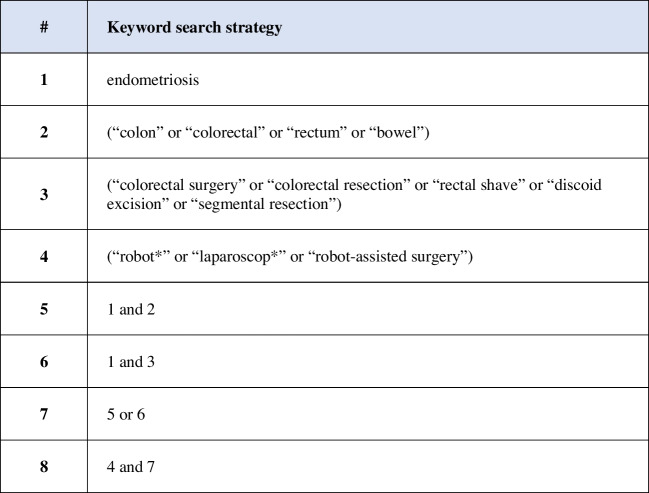
PRISMA diagram

The steps for identifying studies for this review were summarized in the PRISMA diagram (Fig. 1). The initial search resulted in 223 studies. After the removal of duplicates, 192 studies underwent title and abstract screening, leaving 25 full-text studies to be reviewed. A further 14 were excluded for reasons highlighted in Fig. 1, leaving 11 studies [20–30] to be included in the review.

### Study characteristics

Table 2 compiles the data describing the studies’ characteristics. In summary, the study range spanned from 2012 to 2022. Among these studies, nine studies [20–28] were non-comparative case series focusing on RALS, whereas two studies [29, 30] were comparative cohort studies. Overall, the studies included 527 participants, of which 451 received surgical treatment for bowel DE (which may have also included concurrent removal of other sites of disease)—consisting of 368 RALS and 83 SLS participants. All participants who had treatment for DE which did not involve bowel were excluded.

**Table 2:** Characteristics of studies evaluating robotic surgery for the treatment of bowel endometriosis

Author	Year	Country	Study Type	Data collection	Group	Sample size	Procedures of interest	Key conclusion
Ercoli et al. [20]	2012	Italy	CS	Retrospective	RALS	22	RS, SR	Use of RALS for bowel DE treatment is feasible and safe.
Cassini et al. [21]	2013	Italy	CS	Retrospective	RALS	19	SR	Use of RALS for bowel DE treatment is feasible and safe.
Collinet et al. [22]	2014	Multinational^a^	CS	Retrospective	RALS Others^b^	92 72	RS, SR	RALS is feasible in the surgical treatment of stage 4 DE.
Siesto et al. [23]	2014	Italy	CS	Retrospective	RALS Others^b^	42 1	RS, SR	Use of RALS for DE is feasible and safe.
Diguisto et al. [24]	2015	France	CS	Retrospective	RALS	28	RS, SR	SR with RALS for treatment of DE is associated with longer OT and LOS when compared to RS.
Pellegrino et al. [25]	2015	Italy	CS	Prospective	RALS	25	RS	RS with RALS for bowel DE treatment is feasible and safe.
Morelli et al. [26]	2016	Italy	CS	Prospective	RALS	10	SR	RALS for bowel DE treatment is associated with low risks of complication and preservation of urinary and sexual function.
Abo et al. [27]	2017	France	CS	Prospective	RALS Others^b^	32 3	RS, DE, SR	Use of RALS for bowel DE treatment is feasible and safe, but not superior to SLS.
Graham et al. [28]	2019	USA	CS	Retrospective	RALS	15	RS, SR	Use of RALS for treatment of bowel DE is feasible and safe.
Raimondo et al. [29]	2021	Italy	Co	Prospective	RALS SLS	22 22	RS, DE, SR	Similar operative outcomes between RALS and SLS for bowel discoid excision, except for longer operative room time.
Ferrier et al. [30]	2022	France	Co	Prospective	RALS SLS	61 61	RS, DE, SR	RALS provides lower rates of intraoperative complications for discoid excision or SR procedures when compared to SLS.

^a^Belgium, England, France, Italy, Japan

^b^Indicate patient groups with endometrial involvement in sites other than bowel (e.g., bladder, ureter)

*CS* case series, *Co* cohort studies, *RALS* robotic-assisted laparoscopic surgery, *SLS* standard laparoscopic surgery, *RS* rectal shaves, *DE* deep infiltrating endometriosis, *SR* segmental resection, *OT* operating time, *LOS* length of hospital stay

### Surgical procedural details

Nine studies reported the utilization of the Da Vinci System (Intuitive Surgical, Inc.) as their robotic platform (Table 3), with Ercoli et al. likely to have also used this system in 2010. Where patient positioning was described, most were positioned in lithotomy, a configuration commonly used in gynecological, rectal and urological procedures [31]. The depictions of port placements were mostly consistent with what has been described in existing literature [11]. For the two comparative studies [29, 30], the assignment of patients into either the RALS or SLS approach did not follow a formal randomization process, as it solely depended on the availability of the robotic theatre and equipment.

**Table 3:** Surgical system set-up and operative indication of studies evaluating robotic surgery for the treatment of bowel endometriosis

Author	Robotic system	Patient position	Number of ports and placement	Endometriotic lesion indications		
				Rectal shaving	Discoid excision	Segmental resection
Ercoli et al. [20]	NR	NR	- Three 8-mm robotic ports: umbilicus, R IF, L IF - 12-mm assistant port: R subcostal - 5-mm assistant port: L subcostal	Lesions limited to the bowel serosa	N/A	Infiltration into the rectal mucosa and the presence of more than one intestinal stricture
Cassini et al. [21]	da Vinci S/Si	Modified lithotomy	- 12-mm camera port: umbilicus - Two 8-mm robotic ports: R IF, L IF - 1-mm stapler port: R subcostal - 5-mm additional port: NR	N/A	N/A	Lesions > 2 cm infiltrating the bowel, or presence of multifocal lesions, or circumferential involvement of > 50%
Collinet et al. [22]	da Vinci S/Si	NR	- Three to four robotic ports: NR	NR	N/A	NR
Siesto et al. [23]	da Vinci S	Lithotomy	- 12-mm port: umbilicus - Two 8-mm robotic ports: R IF, L IF - 10-mm port: L subcostal	Lesions limited to the bowel serosa	N/A	Lesions with infiltrations into layers deeper than the serosa (muscularis and mucosa layer)
Diguisto et al. [24]	da Vinci S	Lithotomy	NR	Unifocal lesions not involving the mucosal layer.	N/A	Multifocal or extensive lesion with mucosal involvement
Pellegrino et al. [25]	da Vinci S	Lithotomy	- 12-mm port: umbilical incision - Two 8-mm robotic ports: R IF, L IF - 10-mm port: L subcostal	Superficial lesions without full-thickness bowel wall infiltration	N/A	N/A
Morelli et al. [26]	da Vinci Si	Modified lithotomy	- 12-mm optical port: R of umbilicus - Two 8-mm robotic ports: R IF, L IF - 1-2mm port: suprapubic - 10mm port: R subcostal	N/A	N/A	Lesions involving the bowel muscularis layer
Abo et al. [27]	da Vinci S/Si	Modified lithotomy	- Three 8-mm robotic ports: left of umbilicus, R IF, and L IF - 10-mm assistant port: R IF	NR	NR	Multifocal lesions causing severe bowel stenosis, or an area at risk of postoperative necrosis
Graham et al. [28]	da Vinci Si	Lithotomy	- 12-mm port: umbilicus - Two 8-mm ports: R IF and L IF - 5-mm assistant port: L subcostal	Lesions < 1 cm or involving < 30% of the bowel circumference	N/A	Lesions involving > 30% of the circumference of the bowel lumen
Raimondo et al. [29]	da Vinci Si/Xi	NR	NR	Attempted first for all cases	If macroscopic residual disease were observed after rectal shaving	
Ferrier et al. [30]	NR	NR	- Three 8-mm ports: umbilicus, R IF, L IF - 5-mm assistant port: NR - 12-mm port: R abdomen	Superficial lesions without involvement of the mucosal layer	Lesions involving the muscularis, ≤ 3cm or < 90° of the bowel circumference	Multifocal lesions > 3cm in length and involving > 90° of the bowel circumference

*L* left, *R* right, *IF* iliac fossa, *NR* not reported, *N/A* not applicable to the study

**Fig. 2:**
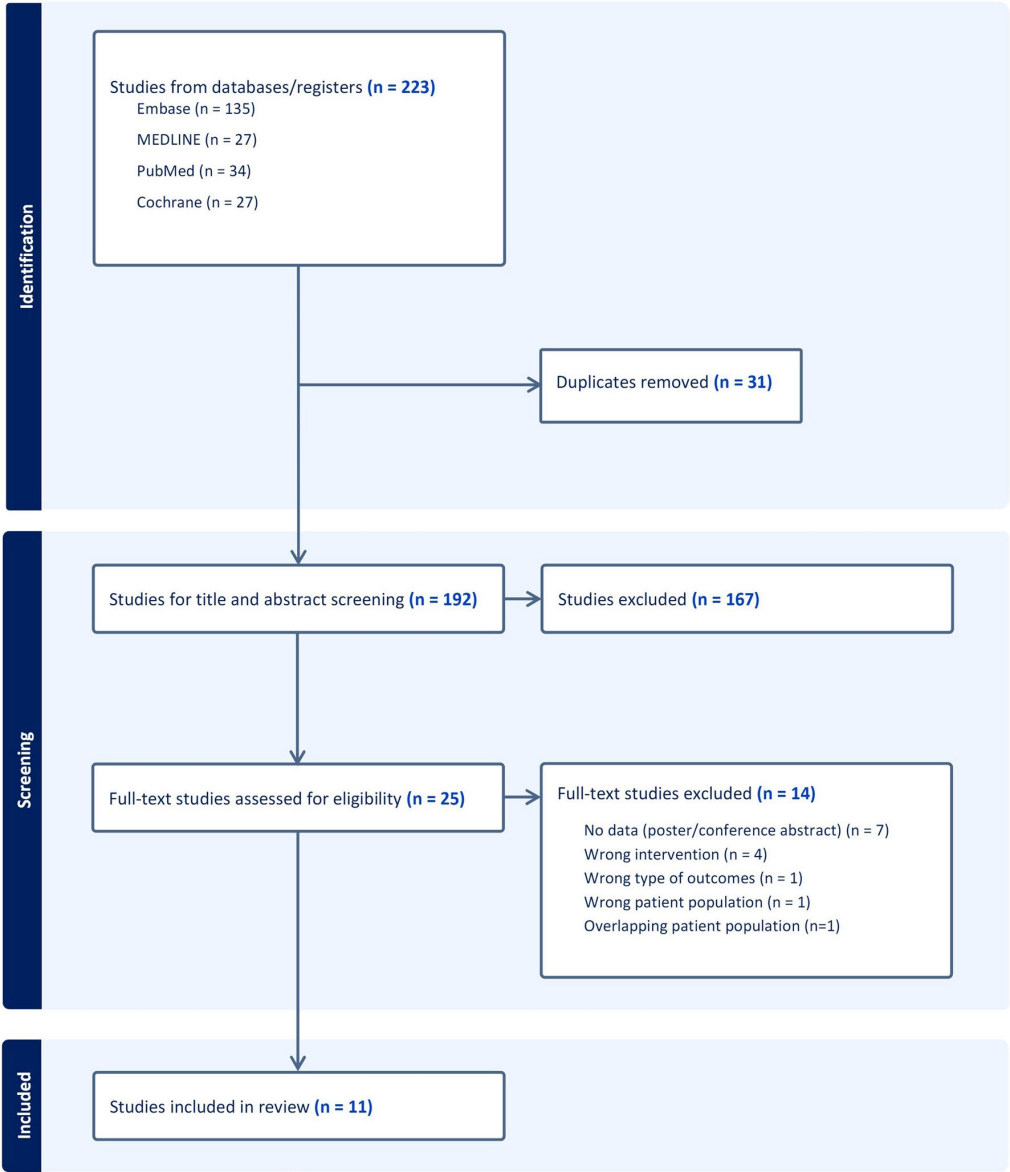
Proportion of RALS and SLS participants stratified by surgical techniques. Data presented in n (%). RALS, robotic-assisted laparoscopic surgery; SLS, Standard laparoscopic surgery; *DE*: Deep infiltrative endometriosis; *RS*: Rectal shaves; *SR*: Segmental resection

In addition, all studies provided insight into at least one of the following techniques: rectal shaves, discoid excisions and segmental resections. Most studies provided indications regarding the characteristics of endometrial lesions for each procedure (Table 3). However, the reporting of indications was not standardized, as each paper puts forward its own distinct criteria. As depicted in Fig. 2, among the participants who underwent RALS and SLS for bowel DE, 59% and 35% had shaves, 8% and 19% had discoid excisions, and 33% and 42% had segmental resections.

### Surgeon experience

When interpreting the results from these studies, it is important to consider the surgical experience, particularly in the RALS group, as many of these studies may represent the surgical learning curve of this new technology.

There was significant heterogeneity in the disclosure and experience of surgeons participating in these studies, as summarized in Table 4, but a trend towards more explicit disclosure of surgical robotic experience can be seen in more recent studies. As expected, all surgeons involved were also trained in laparoscopic surgery.

**Table 4:** Summary of surgeon experience in performing RALS

Author	Year	Surgeon experience
Ercoli et al. [20]	2012	Undisclosed
Cassini et al. [21]	2013	Undisclosed Learning curve acknowledged in discussion Hybrid laparoscopic approach taken in segmental resections, where standard laparoscopy used to mobilise and anastamose.
Collinet et al. [22]	2014	Gynaecologic surgeons trained in laparoscopic surgery
Siesto et al. [23]	2014	Single surgeon with extensive experience in laparoscopic and abdominal gynecological surgery
Diguisto et al. [24]	2015	Undisclosed
Pellegrino et al. [25]	2015	Single surgeon with extensive laparoscopic experience
Morelli et al. [26]	2016	Experienced minimally invasive surgeons, performed > 900 cases.
Abo et al. [27]	2017	Senior gynaecologic surgeon who could perform rectal shaves and ureterolysis. Only 35 of > 250 cases managed robotically in 3 years Learning curve acknowledged in discussion
Graham et al. [28]	2019	Surgeons who had performed fellowships in robotic surgery, having performed > 500 robotic cases.
Raimondo et al. [29]	2021	Senior surgeons trained in minimally invasive surgery, having performed > 25 robotic cases
Ferrier et al. [30]	2022	Experienced gynaecological surgeons trained in minimally invasive surgery, who would perform both RALS and SLS

### Operative and postoperative parameters

The operative and postoperative findings from each study were compiled in Table 5. Most studies reported these metrics using means and standard deviations [33] or medians and ranges. However, a few studies either omitted information on some outcomes or reported means/medians without corresponding SD/ranges, resulting in their exclusion from calculations done for Table 6. Regarding patient characteristics, the groups exhibited comparable age and body mass index profiles (Table 6). The mean operating times was 227 ± 104 in the RALS group and 171 ± 76 min in the SLS group (*p* < 0.01). For the remaining intraoperative metrics, there were no significant differences in blood loss, intraoperative complications and laparotomy conversions.

**Table 5:** Operative and postoperative findings of studies evaluating robotic surgery for the treatment of bowel endometriosis

Author	Group	Sample size	Age (years)	BMI (kg/m^2^)	Operating time (min)	Blood loss [32]	Laparotomy conversion *n* (%)	IOC *n* (%)	POC *n* (%)	LOS (days)
Ercoli et al. [20]	RALS	22	38 [25–45]	21 [17–25]	370 [260–720]^a^ 280 [220–365]^b^	100 [50–250]^a^ 200 [100–350]^b^	0 (0)	0 (0)	1 (4.5)	8 [6–10]^a^ 5 [4–7]^b^
Cassini et al. [21]	RALS	19	37 [25–45]	21 [17–25]	370 (250–720)	150 [50–350]	0 (0)	0 (0)	2 (11)	5 [3–8]
Collinet et al. [22]	RALS	92	34.1 (7.3)	24.4 (8.2)	188.2 (75.7)	127.5 (293.3)	1 (1.1)	4 (4.3)	NR	4.2 (2.7)
Siesto et al. [23]	RALS	42	34 [27.3–45.5]	22.3 [17.2–41]	200 [57–366]	120 [100–1000]	1 (2.4)	0 (0)	2 (4.8)	3 [2–8]
Diguisto et al. [24]	RALS	28	34.1 (2.1)	24.1 (1.1)	190 (51)	274 (51)	1 (3.6)	2 (7.1)	NR	4.9 (51)
Pellegrino et al. [25]	RALS	25	33.9 (6.1)	21.2 (2.9)	174 [75–300]	0 [0–100]	0 (0)	1 (4)	0 (0)	3 [2–4]
Morelli et al. [26]	RALS	10	36.5 [28–47]	22.4 [18.2–25.9]	280 [180–240]	200 [100–400]	0 (0)	0 (0)	1 (10)	6 [4–7]
Abo et al. [27]	RALS	32	36 (8.4)	24.5 (5.9)	207 (67)	NR	0 (0)	NR	3 (9.4)	NR
Graham et al. [28]	RALS	15	38 (2)	29 (2.1)	342 (42.7)	283 (91.6)	0 (0)	0 (0)	5 (33)	2 (1.5)
Raimondo et al. [29]	RALS SLS	22 22	38 (7) 36 (5)	24.5 [21–27] 22.5 [21–24]	207 (79) 177 (63)	184 (214) 144 (101)	1 (4.5) 0 (0)	1 (4.5) 0 (0)	4 (18) 1 (4.5)	8 (7) 6 (2)
Ferrier et al. [30]	RALS SLS	61 61	36 (7) 35 (7)	25 (5) 26 (8)	208 (90) 169 (81)	161 (141) 188 (266)	2 (3.3) 1 (1.6)	2 (3.3) 6 (9.8)	20 (33) 21 (34)	7.5 (3.9) 7.8 (4.6)

Data are presented either in mean [33], median [range] or *n* (%)

Ercoli et al. presented separate outcomes for two RALS subgroups: rectal shaves (*n* = 10)^a^ and segmental resections (*n* = 12)^b^

*RALS* robotic-assisted laparoscopic surgery, *SLS* standard laparoscopic surgery, *BMI* body mass index, *IOC* intraoperative complications, *POC* postoperative complications, *NR* not reported

**Table 6:** Comparative analysis of RALS and SLS patient groups

	RALS (*n = 368*)	SLS (*n = 83*)	*p* value
Age (years)	35.8 ± 6.1	35.3 ± 6.5	0.52
BMI (kg/m^2^)	24.5 ± 5.7	25.3 ± 6.9	0.33
Surgical procedure			
Rectal shaving	217 (59%)	29 (35%)	
Discoid excision	30 (8%)	19 (23%)	
Segmental resection	121 (33%)	35 (42%)	
Operative parameters			
Operating time (min)	227 ± 104^a^	171 ± 76	< 0.01
Intraoperative complications	10 (3.0%)^b^	6 (7.2%)	0.07
Conversion to laparotomy	6 (1.6%)	1 (1.2%)	0.78
Blood loss [32]	155 ± 207^c^	176 ± 234	0.46
Post-operative outcomes			
Duration of hospital stay (days)	5.1 ± 3.6^c^	7.3 ± 4.1	< 0.01
Grade of complications (Clavien-Dindo Grade)			
Major complications (III–IV)	15 (4.4%)^a^	3 (3.6%)	
Minor complications (I–II)	22 (6.4%)^a^	19 (22.9%)	

Data presented either in mean ± SD or *n* (%)

^a^*n* = 340; ^b^*n* = 336; ^c^*n* = 308; sample size varies due to studies excluded for not reporting outcomes

*RALS* robotic-assisted laparoscopic surgery, *SLS* standard laparoscopic surgery, *BMI* Body mass index

Regarding postoperative outcomes, the mean hospital stay was 5.1 ± 3.6 days in the robotic group and 7.3 ± 4.1 days in the standard group (*p* < 0.01). Postoperative complications were stratified based on the Clavien-Dindo classification system to grade adverse events occurring after a surgical procedure [34, 35]. The classification categorizes complications into grades ranging from I to V, in increasing severity. For the purposes of this study, complications were grouped into minor (I–II) and major (III–IV) complications, excluding grade V (death) as it was not reported in any studies. Overall, major and minor complications occurred in 4.4% and 6.4% of cases in the robotic group, compared to 3.6% and 22.9% in the standard group. Anastamotic leak rate of 0.6% in the RALS group (*n* = 2) lies within acceptable limits. It is important to note that as the overall number of patients were low, none of these differences reached statistical significance.

From a functional perspective, 4 studies reported significant improvements in dysmenorrhoea, dyspareunia, dyschezia and chronic pelvic pain within the RALS group at least 6 months post operatively [20, 21, 25, 29] according to the 10 point VAS scale. Raimondo et al. also found an equal improvement when compared to SLS [29]. Morelli et al. found an improvement in quality of life at 6 months [26] and Abo et al. found improvements in bowel function up to 24 months post operatively [27]. However, the heterogeneity of these measures precluded statistical analysis within the context of this systematic review.

## Discussion

Minimally invasive laparoscopy has been the standard in the surgical treatment of endometriosis, including deep infiltrative lesions of the bowel [1, 6, 9]. However, there are known constraints to standard laparoscopy, which include the loss of three-dimensional visualization, reduced degrees of movement and the fulcrum effect [36]. The introduction of robotic surgical platforms aimed to address some of these limitations [36, 37]. While meta-analyses [38, 39] established the feasibility of robotic surgery as an alternative to standard laparoscopy for treating endometriosis, none addressed it in the context of bowel DE. A review by Hur and Falcone [11] had a similar focus, providing a general overview of the role of RALS in bowel endometriosis. However, this present study placed an additional emphasis on conducting a comprehensive review in accordance with the PRISMA guidelines and comparative analysis of data using descriptive statistics.

### Choice of surgical techniques using RALS

Significant differences in the choice of surgical techniques emerged between RALS and SLS, with rectal shaves more frequently utilized with RALS (Table 6). The higher prevalence of shaves in the robotic group could be attributed to the benefits associated with robot-assisted surgery, which offers better vision to aid in assessment of disease depth, allowing surgeons to perform complete excisions with clear margins with rectal shaves, and improved dexterity enabling easier and secure suturing within the confines of the pelvis. When more extensive resection is performed, the technical complexities involved may diminish some of the advantages offered by robotic surgery.

Another factor to consider is the inherent variation of surgical indications described in the studies. The choice of technique itself is dependent upon several factors, including the extent and depth of bowel involvement [6, 9–11]. This inherent variability in decision-making reflects the multitude of factors which influence the personalized nature of treatment decisions.

The decision to perform segmental resections balance a lower recurrence risk against the increased risk of major complications, particularly anastomotic leak and requirement for stoma. In the cohort of patients reviewed, 2 anastomotic leaks were identified, both within the RALS group. This could be explained by a number of factors. Sampling bias is likely to have played a significant role in this finding, particularly in the SLS group, where only 35 patients underwent segmental resection. A large multicenter meta-analysis, including 1622 patients puts this risk at 1.9% [40]. Comparatively, the rate of 0.6% in the RALS group is within acceptable limits. Furthermore, as these cohort studies are unblinded, selection bias may be present, where more complex cases were undertaken when the robotic system is available, as acknowledged by Graham et al. [28].

However, the increased prevalence of segmental resections within the SLS cohort itself introduces a potential confounding variable, given its association with prolonged surgical duration and an increased risk of postoperative complications [41, 42]. These factors can naturally influence other surgical outcomes, such as recovery times and complication rates.

### Operative and postoperative outcomes of RALS

This review suggests that RALS is feasible and safe for treating symptomatic patients with bowel deep infiltrating endometriosis, leading to minimal surgical complications and infrequent conversion to laparotomy. Additionally, there is a trend towards fewer intraoperative complications in the robotic group, although this has not reached clinical significance (Table 6). One study [26] even associates RALS with the preservation of urinary function and sexual well-being up to a year post-surgery. Intraoperative blood loss, another important metric influencing surgical complications, was likewise found to exhibit no significant differences between the groups. The studies included in this review indicate that RALS confers at least an equal benefit in terms of resolution of preoperative symptoms to SLS, but true statistical analysis was not possible due to the heterogeneity of the data.

However, when compared to standard laparoscopy, RALS is associated with significantly longer operating duration, consistent with other reports within the existing literature. Numerous reviews and randomized trials have explored the differences in operating times between RALS and SLS for various subsets of endometriosis patients [38, 39, 43]. While many of these studies reached similar conclusions regarding extended operating times with RALS, there were notable variations in the reported data. Factors contributing to these variations include the selective exclusion of patients with bowel involvement or inclusions of superficial endometriosis in these studies, leading to shorter reported operating times. This complexity underscores the importance of considering specific study contexts and inclusion criteria when interpreting data on operating times.

Postoperatively, the robotic group exhibits a shorter hospital stay, with differences in postoperative complication rates not reaching statistical significance (Table 6). This is likely attributable to a smaller sample size, as compared to larger studies (368 and 83 vs 4721) [44]. Furthermore, many of the included studies acknowledged the learning curve experienced in performing robotic surgery, compared to their significant laparoscopic experience.

In addition, it is important to note that the observational nature of these included studies inherently provides evidence of lower quality compared to RCTs [45]. These studies, due to the lack of controlled interventions, have limitations in their ability to establish causal relationships.

### Translation into clinical practice

Prolonged operating time can largely be attributed to the unique characteristics of robotic surgery, with additional time required for tasks such as docking, robot positioning and troubleshooting technical issues [11, 36]. Previous research [46] describing surgical treatments of advanced endometriosis had shown that longer durations of surgery potentially increase the physiological stress on the patient, adversely affecting recovery times and postoperative outcomes.

Another factor to consider is that operating room time is a valuable resource in healthcare. Prolonged surgeries consume more resources, including personnel, equipment and facilities—on top of the costs incurred for implementing and maintaining the robotic equipment [47]. Consequently, this can result in higher immediate costs, which may be offset by the significantly shorter length of stay and lower rates of surgical complications. Furthermore, as the use of the robotic systems mature, it is expected that the operating time will decrease as the learning curve is overcome by both the surgeons and their supporting theatre staff.

In summary, from a practical perspective, the availability of a robotic system can pose a barrier to the adoption of RALS due to its high start-up costs. Nevertheless, as demonstrated in this review, there are potential benefits to using RALS which can only be fully understood through increased availability and utilization of robotic operating systems within the healthcare system. It is important to note that comprehensive cost-benefit analyses specific to RALS in treating bowel DE have been lacking thus far. Future research is essential to provide additional perspectives on the medico-economic implications associated with RALS.

### IDEAL staging of RALS for bowel DE

The concept of the IDEAL framework was developed in 2009 by McCulloch et al. to evaluate new and emerging surgical innovation. The life cycle of an innovation is described in 5 stages; Idea, Development, Exploration, Assessment and Long-term Study. Criteria for these 5 stages were developed to evaluate the safety, efficacy and effectiveness of an innovation, and were derived from a Delphi survey, expert workshop and discussions at IDEAL conferences held in Oxford (2016) and New York (2017), with ongoing conferences to update these recommendations [12–14].

The evaluation of RALS for the treatment of bowel DE reveals a dynamic landscape of evolving surgical techniques and research methodologies as evidenced by the studies highlighted in this review. In the early 2010s, studies [20–28] on this topic predominantly featured single-centre and/or retrospective case series, indicative of the IDEAL Stage 2a (Development) [48]. These studies sought to establish the viability and safety of RALS for bowel DE, along with building consensus on the technical aspects of the procedure and sharing early clinical experiences.

However, recent papers [29, 30] on the technique have transitioned into Stage 2b (Exploration), characterized by prospective, multicentric cohort comparative studies. This evolution reflects the maturity of the technique, supported by robust findings indicating its feasibility and safety, and is summarised in Table 7. Studies directly comparing RALS against standard laparoscopy are emerging, shedding light on the learning curves associated with RALS and highlighting the necessity for RCTs to provide more definitive evidence. Based on these considerations, the overall staging of RALS for bowel DE can be classified as the endpoint of stage 2b. This signifies a consensus on the maturity of the technique, favourable clinical outcomes and an understanding of desirable outcome measures [48].

**Table 7:** Studies associated with IDEAL Stages

IDEAL Stage	Studies investigating treatment of bowel DE with RALS
Stage 2a (Development)	Ercoli 2012 [20] Cassini 2013 [21] Collinet 2014 [22] Siesto 2015 [23] Diguisto 2015 [24] Pellegrino 2015 [25] Morelli 2016 [26] Abo 2017 [27] Graham 2019 [28]
Single Center Single Intervention Case Series Prospective Cohort	
Stage 2b (Exploration) Bridge from observational to comparative evaluation Prospective multi-center exploration cohort studies	Raimondo 2021 [29] Ferrier 2022 [30]

Looking ahead, it is imperative that multicentre RCTs be undertaken to provide higher-quality evidence to investigate the comparative effectiveness of RALS versus SLS. Notably, the upcoming ROBEndo RCT [49] represents a promising step in this direction and holds the potential to influence future research in this field.

Furthermore, the lack of studies reporting on more specific outcomes such as urinary and sexual function, cost analysis and fertility means there are many avenues to explore for further research as the safety profile of RALS becomes increasingly established.

### Limitations

It is important to recognize that like any literature review, this analysis may be susceptible to evidence selection, reporting and publication biases, as well as the inherent biases of the studies previously mentioned [50]. To limit the sources of these biases, several measures were put in place. Firstly, two independent reviewers performed a thorough assessment of the methodology and risk of bias in all the studies (Tables 8 and 9). Additionally, strict adherence to PRISMA guidelines ensured a structured review and reporting process.

**Table 8:** JBI checklist for case series [16]

	Ercoli et al. [20]	Cassini et al. [21]	Collinet et al. [22]	Siesto et al. [23]	Diguisto et al. [24]	Pellegrino et al. [25]	Morelli et al. [26]	Abo et al. [27]	Graham et al. [28]
Were there clear criteria for inclusion in the case series?	Y	Y	Y	Y	Y	Y	Y	Y	Y
Was the condition measured in a standard, reliable way for all participants included in the case series?	Y	Y	Y	Y	Y	Y	Y	Y	Y
Were valid methods used for identification of the condition for all participants included in the case series?	Y	Y	Y	Y	Y	Y	Y	Y	Y
Did the case series have consecutive inclusion of participants?	Y	Y	Unk.	Y	Y	Y	Y	Y	Y
Did the case series have complete inclusion of participants?	Y	Y	Unk.	Y	Y	Y	Y	Y	Y
Was there clear reporting of the demographics of the participants in the study?	Y	Y	Y	Y	Y	Y	Y	Y	Y
Was there clear reporting of clinical information of the participants?	Y	Y	Y	Y	Y	Y	Y	Y	Y
Were the outcomes or follow up results of cases clearly reported?	Y	Y	Y	Y	Y	Y	Y	Y	Y
Was there clear reporting of the presenting site(s)/clinic(s) demographic information?	N	N	N	N	N	N	N	N	N
Was statistical analysis appropriate?	Y	Y	Y	Y	Y	Y	Y	Y	Y

*Y* Yes; *N* No; *N/A* Not applicable to study; *Unk* Unknown

**Table 9:** JBI checklist for cohort studies [17]

	Raimondo et al. [29]	Ferrier et al. [30]
Were the two groups similar and recruited from the same population?	Y	Y
Were the exposures measured similarly to assign people to both exposed and unexposed groups?	Y	Y
Was the exposure measured in a valid and reliable way?	Y	Y
Were the confounding factors identified?	Y	Y
Were strategies to deal with confounding factors stated?	Y	Y
Were the groups/participants free of the outcome at the start of the study (or at the moment of exposure)?	N/A	N/A
Were the outcomes measured in a valid and reliable way?	Y	Y
Was the follow up time reported and sufficient to be long enough for outcomes to occur?	Y	Y
Was follow up complete, and if not, were the reasons to loss to follow up described and explored?	Y	Y
Were strategies to address incomplete follow up utilized?	N/A	N/A
Was appropriate statistical analysis used?	Y	Y

*Y* Yes; *N* No; *N/A* Not applicable to study

However, the nature of the included studies inherently provides lower-quality evidence compared to prospective RCTs [45]. While these studies offer valuable insights into real-world clinical practices, the observational nature and lack of controlled interventions limit their ability to establish definitive causal relationships. Furthermore, their vulnerability to confounders presents a concern, given that this review cannot address all potential factors that might influence the outcomes, such as the surgeons’ learning curve for RALS.

There was significant heterogeneity in the studies available within the current research landscape, which precluded the performance of a formal meta-analysis. Although we performed statistical analyses which included comparable factors from these studies, the findings should be interpreted with these limitations in mind.

## Conclusion

Robotic-assisted laparoscopic surgery has emerged as an alternative to conventional laparoscopy in the context of deep infiltrative endometriosis involving the bowel. Our review suggests that RALS offers potential benefits of reduced postoperative hospital stay and a trend towards lower intraoperative complications, with longer operating times. As surgeons and techniques mature, well-designed randomized studies are imperative to further define the safety and efficacy of RALS in bowel endometriosis, aligning with an IDEAL framework stage of 2b. Future studies should address the impact of the learning curve of robotic surgery, potential differences in resection margins and functional outcomes in DE, as well compare each technique; rectal shave, discoid excision and segmental resection, to define the advantages and disadvantages of each system.

## Supplementary information

ESM 1 (PDF 84 kb)

## Data Availability

No datasets were generated or analysed during the current study.
